# 6-Chloro-*N*-(2-meth­oxy­phen­yl)pyridazin-3-amine

**DOI:** 10.1107/S1600536812001535

**Published:** 2012-01-18

**Authors:** Abdul Qayyum Ather, M. Nawaz Tahir, Muhammad Naeem Khan, Misbahul Ain Khan, Muhammad Makshoof Athar

**Affiliations:** a Department of Chemistry, Islamia University, Bahawalpur, Pakistan; b Applied Chemistry Research Center, PCSIR Laboratories complex, Lahore 54600, Pakistan; c University of Sargodha, Department of Physics, Sargodha, Pakistan; d Institute of Chemistry, University of the Punjab, Lahore, Pakistan

## Abstract

The asymmetric unit of the title compound, C_11_H_10_ClN_3_O, contains two geometrically different mol­ecules, *A* and *B*, in both of which the pyridazine rings are essentially planar with r.m.s. deviations of 0.0137 and 0.0056Å, respectively. In mol­ecule *A*, the dihedral angle between the pyridazine and benzene rings is 6.5 (2)°, whereas in mol­ecule *B* it is 27.93 (7)°. In mol­ecule *B*, an intramolecular N—H⋯O hydrogen bond forms an *S*(5) ring motif. In both molecules, *S*(6) ring motifs are present due to non-classical C—H⋯N hydrogen bonds. The π–π inter­actions between the pyridazine rings of *A* mol­ecules [3.4740 (13) Å] and *B* mol­ecules [3.4786 (17) Å] have very similar centroid–centroid separations. π–π Inter­actions also occur between the benzene rings of *B* mol­ecules with a centroid–centroid separation of 3.676 (2) Å and a slippage of 1.02 Å. In the crystal, the mol­ecules are linked into chains extending along [010] by C—H⋯N and C—H⋯Cl interactions.

## Related literature

For general background and related structures, see: Ather *et al.* (2010**a* ▶,*b* ▶,c* ▶; 2011 ▶). For graph-set notation, see: Bernstein *et al.* (1995 ▶).
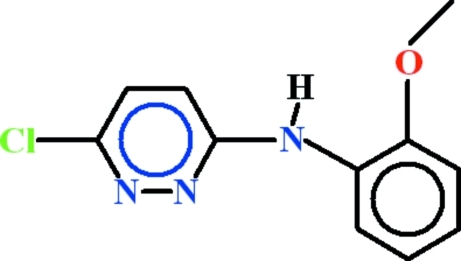


## Experimental

### 

#### Crystal data

C_11_H_10_ClN_3_O
*M* *_r_* = 235.67
Monoclinic, 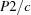

*a* = 14.6018 (5) Å
*b* = 10.8574 (3) Å
*c* = 17.4630 (6) Å
β = 126.438 (2)°
*V* = 2227.29 (14) Å^3^
*Z* = 8
Mo *K*α radiation
μ = 0.32 mm^−1^
*T* = 295 K
0.32 × 0.16 × 0.14 mm

#### Data collection

Bruker Kappa APEXII CCD diffractometer
Absorption correction: multi-scan (*SADABS*; Bruker, 2005 ▶) *T*_min_ = 0.938, *T*_max_ = 0.957
17904 measured reflections
4387 independent reflections
2815 reflections with *I* > 2σ(*I*)
*R*_int_ = 0.027

#### Refinement

*R*[*F* ^2^ > 2σ(*F* ^2^)] = 0.039
*wR*(*F* ^2^) = 0.110
*S* = 1.03
4387 reflections
291 parameters
H-atom parameters constrained
Δρ_max_ = 0.20 e Å^−3^
Δρ_min_ = −0.21 e Å^−3^

### 

Data collection: *APEX2* (Bruker, 2009 ▶); cell refinement: *SAINT* (Bruker, 2009 ▶); data reduction: *SAINT*; program(s) used to solve structure: *SHELXS97* (Sheldrick, 2008 ▶); program(s) used to refine structure: *SHELXL97* (Sheldrick, 2008 ▶); molecular graphics: *ORTEP-3* (Farrugia, 1997 ▶) and *PLATON* (Spek, 2009 ▶); software used to prepare material for publication: *WinGX* (Farrugia, 1999 ▶) and *PLATON*.

## Supplementary Material

Crystal structure: contains datablock(s) global, I. DOI: 10.1107/S1600536812001535/rk2330sup1.cif

Structure factors: contains datablock(s) I. DOI: 10.1107/S1600536812001535/rk2330Isup2.hkl

Supplementary material file. DOI: 10.1107/S1600536812001535/rk2330Isup3.cml

Additional supplementary materials:  crystallographic information; 3D view; checkCIF report

## Figures and Tables

**Table 1:** Hydrogen-bond geometry (Å, °)

*D*—H⋯*A*	*D*—H	H⋯*A*	*D*⋯*A*	*D*—H⋯*A*
N3—H3⋯O1	0.86	2.14	2.579 (3)	111
N3—H3⋯N4	0.86	2.48	3.278 (2)	155
N6—H6*A*⋯N1^i^	0.86	2.44	3.270 (3)	161
C2—H2⋯Cl2^ii^	0.93	2.79	3.526 (2)	137
C3—H3*A*⋯N5	0.93	2.61	3.503 (3)	161
C6—H6⋯N2	0.93	2.31	2.913 (4)	122
C17—H17⋯N5	0.93	2.50	2.992 (3)	113

Symmetry codes: (i) 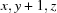; (ii) 
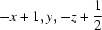
.

**Fig. 1.**
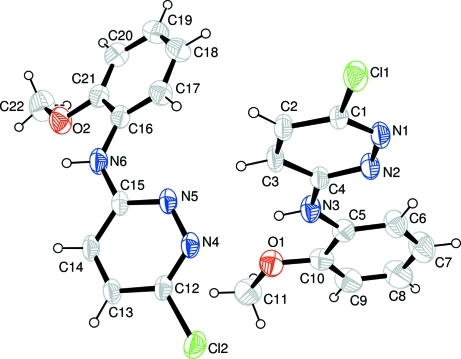
View of the title compound with the atom numbering scheme. The displacement ellipsoids are drawn at the 30% probability level. The H atoms are shown as small spheres of arbitrary radii.

**Fig. 2.**
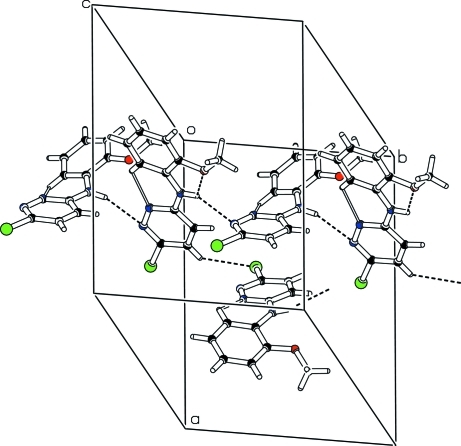
Packing diagram of the title compound showing that molecules form one dimensional polymeric chains along [0 1 0].

## supplementary crystallographic information

### Comment

In continuation to 6-chloropyridazin derivatives (Ather *et al.*, 2010**a*,*b*,c*; 2011), the title compound I (Fig. 1) is being reported here.

The two molecules in the asymmetric unit are present, which differ from each other geometrically. In one molecule, the pyridazin ring A (C1-C4/N1/N2) and the phenyl ring B (C5-C10) are planar with r. m. s. deviation of 0.0137Å and 0.0065Å, respectively. The dihedral angle between A/B is 6.5 (2)°. In second molecule, the pyridazin ring C (C12-C15/N4/N5) and the phenyl ring D (C16-C21) are planar with r. m. s. deviation of 0.0056 and 0.0053Å, respectively and the dihedral angle between C/D is 27.93 (7)°. In the more planar molecule, there exists classical intramolecular H-bonding of N–H···O type (Table 1, Fig. 2) with *S*(5) ring motif (Bernstein *et al.*, 1995). In both molecules *S*(6) ring motifs are formed due to non-classical C–H···N type of H-bondings (Table 1, Fig. 2). The molecules are interlinked due to the H-bondings of C–H···N and C–H···Cl types (Table 1, Fig. 2) to form the one dimensional polymeric chains extending along [0 1 0]. There exist π–π interactions between the centroids of a phenyl and two pyridazin rings with *Cg*A···*Cg*A^i^ = 3.4740 (13)Å, *Cg*C···*Cg*C^i^ = 3.4786 (17)Å and *Cg*D···*Cg*D^ii^ = 3.676 (2)Å (slippage = 1.021Å), where *Cg*A, *Cg*C and *Cg*D are the centroids of the rings A, C and D, respectively. Symmetry codes: (i) 1-*x*, *y*, 1/2-*z*; (ii) -*x*, 1-*y*, -*z*.

### Experimental

An equimolar quantity (6.71 mmol) of 3,6-dichloropyradizine and 2-methoxyaniline in 10 ml of ethanol was heated under reflux for 3 h. The reaction mixture was concentrated under reduced pressure, cooled and poured over 50 ml of distilled water. The precipitate was filtered and dried in oven on 333 K. The dried crude product was recrystallized in ethanol to obtain colourless needles of I.

### Refinement

The H-atoms were positioned geometrically (C–H = 0.93-0.96Å, N–H = 0.86Å) and refined as riding with *U*_iso_(H) = *xU*_eq_(C, N), where *x* = 1.5 for methyl groups and *x* = 1.2 for other H atoms.

### Crystal data

**Table:**

C_11_H_10_ClN_3_O	*F*(000) = 976
*M**_r_* = 235.67	*D*_x_ = 1.406 Mg m^−^^3^
Monoclinic, *P*2/*c*	Mo *K*α radiation, λ = 0.71073 Å
Hall symbol: -P 2yc	Cell parameters from 773 reflections
*a* = 14.6018 (5) Å	θ = 2.4–25.3°
*b* = 10.8574 (3) Å	µ = 0.32 mm^−^^1^
*c* = 17.4630 (6) Å	*T* = 295 K
β = 126.438 (2)°	Needle, colourless
*V* = 2227.29 (14) Å^3^	0.32 × 0.16 × 0.14 mm
*Z* = 8	

### Data collection

**Table:**

Bruker Kappa APEXII CCD diffractometer	4387 independent reflections
Radiation source: fine-focus sealed tube	2815 reflections with *I* > 2σ(*I*)
graphite	*R*_int_ = 0.027
Detector resolution: 8.0 pixels mm^-1^	θ_max_ = 26.0°, θ_min_ = 1.9°
ω scans	*h* = −17→18
Absorption correction: multi-scan (*SADABS*; Bruker, 2005)	*k* = −13→12
*T*_min_ = 0.938, *T*_max_ = 0.957	*l* = −21→21
17904 measured reflections	

### Refinement

**Table:**

Refinement on *F*^2^	Primary atom site location: structure-invariant direct methods
Least-squares matrix: full	Secondary atom site location: difference Fourier map
*R*[*F*^2^ > 2σ(*F*^2^)] = 0.039	Hydrogen site location: inferred from neighbouring sites
*wR*(*F*^2^) = 0.110	H-atom parameters constrained
*S* = 1.03	*w* = 1/[σ^2^(*F*_o_^2^) + (0.048*P*)^2^ + 0.3696*P*] where *P* = (*F*_o_^2^ + 2*F*_c_^2^)/3
4387 reflections	(Δ/σ)_max_ < 0.001
291 parameters	Δρ_max_ = 0.20 e Å^−^^3^
0 restraints	Δρ_min_ = −0.21 e Å^−^^3^

### Special details

**Table:**

Geometry. All s.u.'s (except the s.u. in the dihedral angle between two l.s. planes) are estimated using the full covariance matrix. The cell s.u.'s are taken into account individually in the estimation of s.u.'s in distances, angles and torsion angles; correlations between s.u.'s in cell parameters are only used when they are defined by crystal symmetry. An approximate (isotropic) treatment of cell s.u.'s is used for estimating s.u.'s involving l.s. planes.
Refinement. Refinement of *F*^2^ against ALL reflections. The weighted *R*-factor *wR* and goodness of fit *S* are based on *F*^2^, conventional *R*-factors *R* are based on *F*, with *F* set to zero for negative *F*^2^. The threshold expression of *F*^2^ > σ(*F*^2^) is used only for calculating *R*-factors(gt) *etc*. and is not relevant to the choice of reflections for refinement. *R*-factors based on *F*^2^ are statistically about twice as large as those based on *F*, and *R*-factors based on ALL data will be even larger.

### Fractional atomic coordinates and isotropic or equivalent isotropic displacement parameters (Å^2^)

**Table:**

	*x*	*y*	*z*	*U*_iso_*/*U*_eq_	
Cl1	0.46850 (6)	−0.13866 (5)	0.05144 (5)	0.0738 (2)	
O1	0.24223 (15)	0.23518 (15)	0.32088 (12)	0.0808 (7)	
N1	0.36545 (16)	−0.13820 (14)	0.13098 (13)	0.0619 (6)	
N2	0.32177 (15)	−0.08784 (14)	0.17449 (13)	0.0607 (7)	
N3	0.28781 (15)	0.08591 (15)	0.23280 (13)	0.0659 (7)	
C1	0.40898 (17)	−0.06681 (16)	0.10082 (14)	0.0523 (7)	
C2	0.41035 (19)	0.06066 (17)	0.10578 (15)	0.0626 (8)	
C3	0.3676 (2)	0.11179 (16)	0.14842 (16)	0.0645 (9)	
C4	0.32520 (17)	0.03400 (16)	0.18478 (14)	0.0528 (7)	
C5	0.23884 (17)	0.0334 (2)	0.27383 (15)	0.0622 (8)	
C6	0.2125 (2)	−0.0903 (2)	0.26998 (19)	0.0792 (10)	
C7	0.1651 (3)	−0.1314 (3)	0.3150 (2)	0.1053 (16)	
C8	0.1428 (3)	−0.0507 (3)	0.3617 (2)	0.1073 (14)	
C9	0.1660 (2)	0.0735 (3)	0.36443 (19)	0.0881 (11)	
C10	0.21385 (19)	0.1147 (2)	0.32131 (16)	0.0673 (9)	
C11	0.2238 (3)	0.3246 (3)	0.3706 (2)	0.0938 (11)	
Cl2	0.56300 (6)	0.35556 (5)	0.46367 (4)	0.0777 (2)	
O2	0.08261 (14)	0.73892 (13)	−0.00138 (12)	0.0856 (7)	
N4	0.40277 (16)	0.36064 (13)	0.28154 (14)	0.0582 (7)	
N5	0.32848 (15)	0.41352 (13)	0.19496 (13)	0.0575 (6)	
N6	0.25123 (16)	0.59100 (15)	0.10279 (14)	0.0722 (7)	
C12	0.47012 (18)	0.42988 (16)	0.35550 (15)	0.0537 (7)	
C13	0.47274 (19)	0.55815 (17)	0.35300 (17)	0.0622 (8)	
C14	0.3991 (2)	0.61149 (17)	0.26792 (17)	0.0655 (9)	
C15	0.32556 (18)	0.53634 (16)	0.18843 (16)	0.0550 (8)	
C16	0.15632 (19)	0.54289 (18)	0.01765 (16)	0.0592 (8)	
C17	0.1480 (2)	0.4246 (2)	−0.01534 (18)	0.0705 (9)	
C18	0.0498 (3)	0.3871 (2)	−0.10086 (19)	0.0834 (10)	
C19	−0.0397 (2)	0.4662 (3)	−0.15407 (19)	0.0842 (11)	
C20	−0.0322 (2)	0.5845 (2)	−0.12320 (19)	0.0760 (10)	
C21	0.0649 (2)	0.62309 (19)	−0.03823 (17)	0.0633 (9)	
C22	−0.0100 (3)	0.8233 (3)	−0.0472 (2)	0.1089 (13)	
H2	0.43954	0.10860	0.08075	0.0752*	
H3	0.29570	0.16458	0.23890	0.0791*	
H3A	0.36609	0.19688	0.15370	0.0775*	
H6	0.22645	−0.14566	0.23738	0.0949*	
H7	0.14864	−0.21451	0.31319	0.1261*	
H8	0.11179	−0.07923	0.39190	0.1288*	
H9	0.14920	0.12855	0.39529	0.1058*	
H11A	0.26440	0.30060	0.43607	0.1408*	
H11B	0.14394	0.32969	0.34210	0.1408*	
H11C	0.25074	0.40345	0.36695	0.1408*	
H6A	0.26458	0.66753	0.10045	0.0866*	
H13	0.52290	0.60433	0.40746	0.0747*	
H14	0.39682	0.69672	0.26167	0.0786*	
H17	0.20860	0.37019	0.02014	0.0846*	
H18	0.04463	0.30724	−0.12243	0.1002*	
H19	−0.10576	0.43986	−0.21125	0.1013*	
H20	−0.09290	0.63851	−0.15978	0.0912*	
H22A	−0.07363	0.78708	−0.05266	0.1638*	
H22B	−0.03118	0.84205	−0.10951	0.1638*	
H22C	0.01230	0.89754	−0.01038	0.1638*	

### Atomic displacement parameters (Å^2^)

**Table:**

	*U*^11^	*U*^22^	*U*^33^	*U*^12^	*U*^13^	*U*^23^
Cl1	0.1073 (5)	0.0477 (3)	0.0915 (4)	0.0106 (3)	0.0727 (4)	0.0023 (3)
O1	0.1095 (13)	0.0681 (10)	0.0948 (12)	0.0036 (9)	0.0771 (11)	−0.0063 (9)
N1	0.0869 (13)	0.0357 (8)	0.0751 (12)	−0.0041 (8)	0.0547 (11)	−0.0027 (8)
N2	0.0787 (13)	0.0389 (8)	0.0778 (13)	−0.0060 (8)	0.0537 (11)	−0.0029 (8)
N3	0.0868 (14)	0.0455 (9)	0.0894 (14)	0.0022 (9)	0.0654 (12)	0.0000 (9)
C1	0.0654 (13)	0.0360 (9)	0.0545 (13)	0.0019 (9)	0.0350 (11)	0.0012 (9)
C2	0.0921 (17)	0.0368 (9)	0.0779 (16)	−0.0039 (10)	0.0608 (14)	0.0000 (10)
C3	0.0981 (18)	0.0305 (9)	0.0837 (16)	−0.0042 (10)	0.0642 (15)	−0.0031 (10)
C4	0.0581 (13)	0.0406 (10)	0.0582 (13)	0.0001 (9)	0.0338 (11)	0.0015 (9)
C5	0.0549 (13)	0.0675 (13)	0.0651 (14)	−0.0018 (10)	0.0361 (12)	0.0015 (11)
C6	0.0869 (18)	0.0732 (15)	0.099 (2)	−0.0176 (13)	0.0670 (17)	−0.0098 (14)
C7	0.120 (3)	0.100 (2)	0.129 (3)	−0.0425 (18)	0.092 (2)	−0.0195 (19)
C8	0.121 (2)	0.129 (3)	0.112 (2)	−0.048 (2)	0.091 (2)	−0.026 (2)
C9	0.0869 (19)	0.113 (2)	0.0851 (19)	−0.0225 (16)	0.0623 (17)	−0.0181 (16)
C10	0.0607 (15)	0.0811 (16)	0.0631 (15)	−0.0031 (12)	0.0384 (13)	−0.0040 (12)
C11	0.122 (2)	0.0861 (18)	0.097 (2)	0.0192 (16)	0.0780 (19)	−0.0074 (15)
Cl2	0.1150 (5)	0.0491 (3)	0.0793 (4)	0.0085 (3)	0.0634 (4)	0.0085 (3)
O2	0.0938 (13)	0.0507 (9)	0.0976 (12)	0.0108 (8)	0.0489 (11)	0.0039 (8)
N4	0.0806 (13)	0.0349 (8)	0.0777 (13)	0.0005 (8)	0.0572 (11)	−0.0002 (9)
N5	0.0726 (12)	0.0352 (8)	0.0763 (12)	−0.0019 (8)	0.0505 (11)	−0.0007 (8)
N6	0.0799 (14)	0.0406 (9)	0.0811 (14)	−0.0046 (9)	0.0397 (12)	0.0088 (9)
C12	0.0736 (14)	0.0378 (9)	0.0731 (14)	0.0029 (9)	0.0564 (13)	0.0027 (10)
C13	0.0846 (16)	0.0377 (10)	0.0767 (16)	−0.0088 (10)	0.0546 (14)	−0.0064 (10)
C14	0.0877 (17)	0.0320 (9)	0.0839 (17)	−0.0032 (10)	0.0549 (15)	0.0024 (10)
C15	0.0677 (14)	0.0369 (10)	0.0758 (15)	−0.0039 (9)	0.0510 (13)	0.0006 (10)
C16	0.0677 (15)	0.0510 (11)	0.0696 (15)	−0.0030 (11)	0.0466 (13)	0.0021 (11)
C17	0.0809 (17)	0.0608 (13)	0.0805 (17)	0.0063 (12)	0.0537 (15)	−0.0031 (12)
C18	0.106 (2)	0.0732 (15)	0.0828 (19)	−0.0028 (15)	0.0625 (19)	−0.0188 (14)
C19	0.0857 (19)	0.093 (2)	0.0746 (18)	−0.0053 (16)	0.0480 (16)	−0.0130 (15)
C20	0.0761 (18)	0.0790 (16)	0.0781 (18)	0.0104 (13)	0.0487 (16)	0.0077 (14)
C21	0.0755 (16)	0.0548 (12)	0.0738 (16)	0.0020 (11)	0.0521 (15)	0.0042 (11)
C22	0.116 (2)	0.0680 (16)	0.137 (3)	0.0319 (16)	0.072 (2)	0.0163 (17)

### Geometric parameters (Å, °)

**Table:**

Cl1—C1	1.731 (3)	C2—H2	0.9300
Cl2—C12	1.737 (2)	C3—H3A	0.9300
O1—C10	1.374 (3)	C6—H6	0.9300
O1—C11	1.431 (4)	C7—H7	0.9300
O2—C21	1.366 (3)	C8—H8	0.9300
O2—C22	1.422 (4)	C9—H9	0.9300
N1—N2	1.363 (3)	C11—H11B	0.9600
N1—C1	1.296 (3)	C11—H11C	0.9600
N2—C4	1.332 (2)	C11—H11A	0.9600
N3—C5	1.399 (4)	C12—C13	1.395 (3)
N3—C4	1.365 (3)	C13—C14	1.342 (3)
N3—H3	0.8600	C14—C15	1.405 (3)
N4—N5	1.358 (3)	C16—C21	1.395 (4)
N4—C12	1.301 (3)	C16—C17	1.383 (3)
N5—C15	1.337 (2)	C17—C18	1.381 (4)
N6—C15	1.356 (3)	C18—C19	1.366 (5)
N6—C16	1.399 (3)	C19—C20	1.372 (4)
N6—H6A	0.8600	C20—C21	1.374 (4)
C1—C2	1.386 (3)	C13—H13	0.9300
C2—C3	1.343 (4)	C14—H14	0.9300
C3—C4	1.402 (4)	C17—H17	0.9300
C5—C10	1.400 (4)	C18—H18	0.9300
C5—C6	1.388 (3)	C19—H19	0.9300
C6—C7	1.394 (5)	C20—H20	0.9300
C7—C8	1.362 (5)	C22—H22A	0.9600
C8—C9	1.384 (5)	C22—H22B	0.9600
C9—C10	1.371 (4)	C22—H22C	0.9600
			
C10—O1—C11	118.5 (3)	H11B—C11—H11C	109.00
C21—O2—C22	118.5 (2)	H11A—C11—H11B	110.00
N2—N1—C1	119.43 (16)	O1—C11—H11B	109.00
N1—N2—C4	118.8 (2)	O1—C11—H11C	109.00
C4—N3—C5	131.21 (18)	H11A—C11—H11C	109.00
C5—N3—H3	114.00	O1—C11—H11A	109.00
C4—N3—H3	114.00	N4—C12—C13	124.4 (2)
N5—N4—C12	119.61 (15)	Cl2—C12—N4	116.94 (14)
N4—N5—C15	118.73 (17)	Cl2—C12—C13	118.68 (17)
C15—N6—C16	130.36 (18)	C12—C13—C14	116.5 (2)
C15—N6—H6A	115.00	C13—C14—C15	118.86 (18)
C16—N6—H6A	115.00	N5—C15—C14	121.9 (2)
N1—C1—C2	124.3 (2)	N6—C15—C14	118.47 (17)
Cl1—C1—C2	119.2 (2)	N5—C15—N6	119.65 (19)
Cl1—C1—N1	116.44 (15)	C17—C16—C21	118.5 (2)
C1—C2—C3	116.9 (2)	N6—C16—C17	125.2 (2)
C2—C3—C4	118.53 (18)	N6—C16—C21	116.29 (19)
N2—C4—C3	121.9 (2)	C16—C17—C18	120.1 (3)
N3—C4—C3	118.34 (17)	C17—C18—C19	120.6 (2)
N2—C4—N3	119.8 (2)	C18—C19—C20	120.0 (3)
N3—C5—C10	116.0 (2)	C19—C20—C21	120.0 (3)
N3—C5—C6	125.5 (2)	C16—C21—C20	120.7 (2)
C6—C5—C10	118.5 (3)	O2—C21—C16	114.2 (2)
C5—C6—C7	119.9 (3)	O2—C21—C20	125.1 (2)
C6—C7—C8	120.5 (3)	C12—C13—H13	122.00
C7—C8—C9	120.5 (4)	C14—C13—H13	122.00
C8—C9—C10	119.5 (3)	C13—C14—H14	121.00
C5—C10—C9	121.1 (2)	C15—C14—H14	121.00
O1—C10—C9	124.6 (3)	C16—C17—H17	120.00
O1—C10—C5	114.3 (2)	C18—C17—H17	120.00
C1—C2—H2	122.00	C17—C18—H18	120.00
C3—C2—H2	122.00	C19—C18—H18	120.00
C4—C3—H3A	121.00	C18—C19—H19	120.00
C2—C3—H3A	121.00	C20—C19—H19	120.00
C7—C6—H6	120.00	C19—C20—H20	120.00
C5—C6—H6	120.00	C21—C20—H20	120.00
C6—C7—H7	120.00	O2—C22—H22A	109.00
C8—C7—H7	120.00	O2—C22—H22B	109.00
C7—C8—H8	120.00	O2—C22—H22C	109.00
C9—C8—H8	120.00	H22A—C22—H22B	109.00
C8—C9—H9	120.00	H22A—C22—H22C	110.00
C10—C9—H9	120.00	H22B—C22—H22C	110.00
			
C11—O1—C10—C5	−177.8 (2)	N3—C5—C6—C7	179.2 (3)
C11—O1—C10—C9	1.7 (4)	C10—C5—C6—C7	−1.7 (4)
C22—O2—C21—C16	−172.8 (3)	N3—C5—C10—O1	−0.4 (3)
C22—O2—C21—C20	8.1 (5)	C6—C5—C10—C9	1.0 (4)
N2—N1—C1—C2	−3.0 (3)	N3—C5—C10—C9	−179.8 (2)
C1—N1—N2—C4	0.0 (3)	C6—C5—C10—O1	−179.6 (2)
N2—N1—C1—Cl1	177.09 (16)	C5—C6—C7—C8	1.0 (5)
N1—N2—C4—C3	2.9 (3)	C6—C7—C8—C9	0.6 (5)
N1—N2—C4—N3	−176.4 (2)	C7—C8—C9—C10	−1.3 (5)
C5—N3—C4—C3	177.8 (2)	C8—C9—C10—O1	−178.9 (3)
C5—N3—C4—N2	−2.9 (4)	C8—C9—C10—C5	0.5 (4)
C4—N3—C5—C6	−3.1 (4)	Cl2—C12—C13—C14	−179.7 (3)
C4—N3—C5—C10	177.8 (2)	N4—C12—C13—C14	0.8 (5)
C12—N4—N5—C15	−1.0 (4)	C12—C13—C14—C15	0.0 (5)
N5—N4—C12—Cl2	−179.8 (2)	C13—C14—C15—N5	−1.3 (5)
N5—N4—C12—C13	−0.3 (5)	C13—C14—C15—N6	179.8 (3)
N4—N5—C15—C14	1.8 (4)	N6—C16—C17—C18	−179.9 (3)
N4—N5—C15—N6	−179.4 (3)	C21—C16—C17—C18	−1.4 (5)
C15—N6—C16—C17	−35.8 (5)	N6—C16—C21—O2	0.8 (4)
C16—N6—C15—N5	14.6 (5)	N6—C16—C21—C20	180.0 (3)
C16—N6—C15—C14	−166.5 (3)	C17—C16—C21—O2	−177.8 (3)
C15—N6—C16—C21	145.7 (3)	C17—C16—C21—C20	1.4 (5)
Cl1—C1—C2—C3	−177.19 (19)	C16—C17—C18—C19	0.5 (6)
N1—C1—C2—C3	2.9 (4)	C17—C18—C19—C20	0.6 (6)
C1—C2—C3—C4	0.1 (4)	C18—C19—C20—C21	−0.7 (5)
C2—C3—C4—N3	176.3 (2)	C19—C20—C21—O2	178.8 (3)
C2—C3—C4—N2	−3.0 (4)	C19—C20—C21—C16	−0.3 (5)

### Hydrogen-bond geometry (Å, °)

**Table:**

*D*—H···*A*	*D*—H	H···*A*	*D*···*A*	*D*—H···*A*
N3—H3···O1	0.86	2.14	2.579 (3)	111
N3—H3···N4	0.86	2.48	3.278 (2)	155
N6—H6A···N1^i^	0.86	2.44	3.270 (3)	161
C2—H2···Cl2^ii^	0.93	2.79	3.526 (2)	137
C3—H3A···N5	0.93	2.61	3.503 (3)	161
C6—H6···N2	0.93	2.31	2.913 (4)	122
C17—H17···N5	0.93	2.50	2.992 (3)	113

Symmetry codes: (i) *x*, *y*+1, *z*; (ii) −*x*+1, *y*, −*z*+1/2.
